# A rare case of endoscopic visualization of a metallic coil in a duodenal ulcer after transcatheter arterial embolization

**DOI:** 10.1093/omcr/omae143

**Published:** 2024-11-25

**Authors:** Prince A Ameyaw, Ans A Jajja, Ysabel Ilagan-Ying

**Affiliations:** Department of Medicine, Bridgeport Hospital/Yale New Haven Health, 267 Grant St, Bridgeport, CT 06610, United States; Department of Medicine, Griffin Hospital, 130 Division St, Derby, CT, United States; Department of Medicine, Section of Digestive Diseases, Yale School of Medicine, 333 Cedar St, New Haven, CT 06510, United States

**Keywords:** upper gastrointestinal bleeding, coil embolization, peptic ulcer disease, esophagogastroduodenoscopy

## Abstract

Transcatheter arterial embolization is the therapy of choice for recurrent peptic ulcer bleeding refractory to standard endoscopic hemostatic techniques. It offers a minimally invasive approach with high efficacy, lower mortality, and complication rates compared to surgery. However, rare adverse events attributed to coil migration including pulmonary embolism, stroke, myocardial infarction, and bowel obstruction have been reported. We report the case of a 72-year-old female with a massive duodenal ulcer bleed refractory to endoscopic hemostatic techniques of epinephrine injection and bipolar cautery. She had a successful transcatheter arterial embolization of the culprit vessels. Repeat esophagogastroduodenoscopy for increasing vasopressor requirements and transfusion unresponsive anemia revealed the visualization of the metallic coil in the duodenal ulcer base with no active bleeding which was successfully managed conservatively through multidisciplinary consultation.

## Introduction

Peptic ulcer bleeding is a life-threatening complication that accounts for up to 60% of all etiologies of acute upper gastrointestinal bleeding [1]. Peptic ulcer disease (PUD) refers to large (greater than 3–5 mm) acid-peptic-induced defects penetrating the submucosa of the digestive tract, predominantly in the stomach and proximal duodenum. PUD has an estimated prevalence of 5%–10% [1], and arises from an imbalance between gastroprotective factors and those that inflict damage to the mucosa including *Helicobacter pylori* infection, nonsteroidal anti-inflammatory drug use, and gastric acid exposure [1]. Management includes risk stratification, volume and restrictive-based transfusion resuscitation, acid suppression, and endoscopic therapies for hemostasis. However, some patients may have recurrent bleeding and failure of endoscopic therapy warranting angiography-based treatment modalities such as transcatheter arterial embolization [2]. Complications of angiography and arterial embolization include access site thrombosis/hemorrhage, contrast reaction and nephropathy, target vessel damage, ischemic injury post-embolization, and coil migration [3]. We present a rare finding of a visible arterial coil at the duodenal ulcer base after embolization for recurrent ulcer bleeding.

## Case report

**Figure 1:**
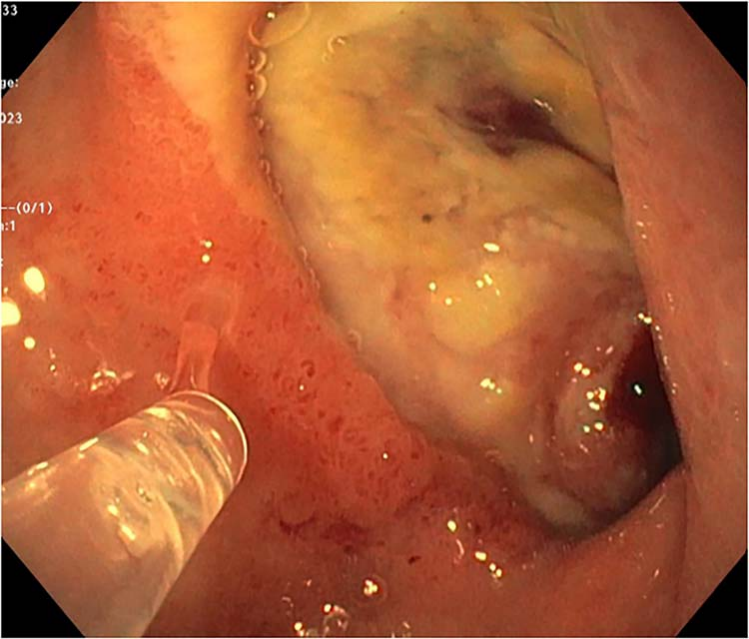
Endoscopic image showing the large duodenal ulcer after achievement of hemostasis with epinephrine injections and bipolar cautery.

**Figure 2:**
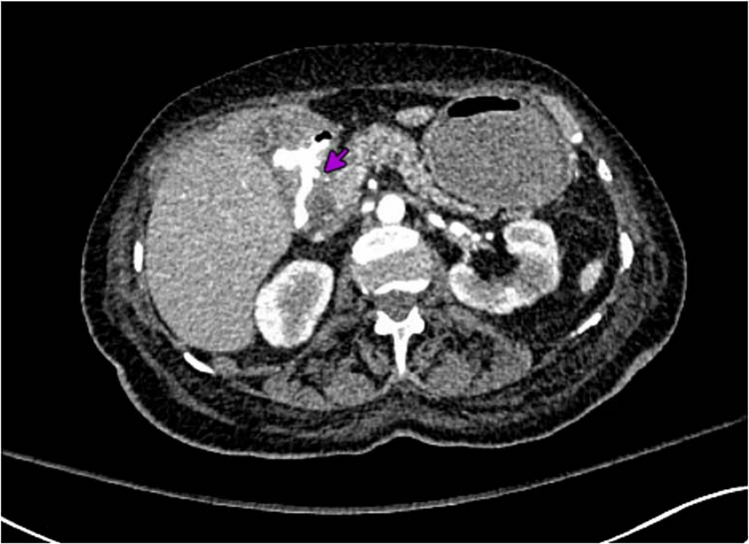
Computed tomography angiography abdomen axial view showing massive arterial bleeding in the first and second portions of the duodenum.

**Figure 3:**
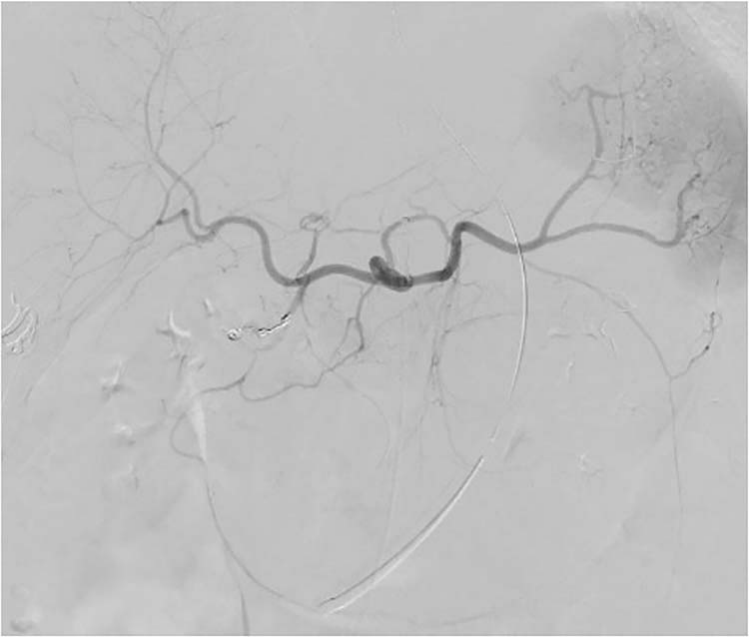
Celiac angiographic view showing embolization coils in a branch of the gastroduodenal artery.

**Figure 4:**
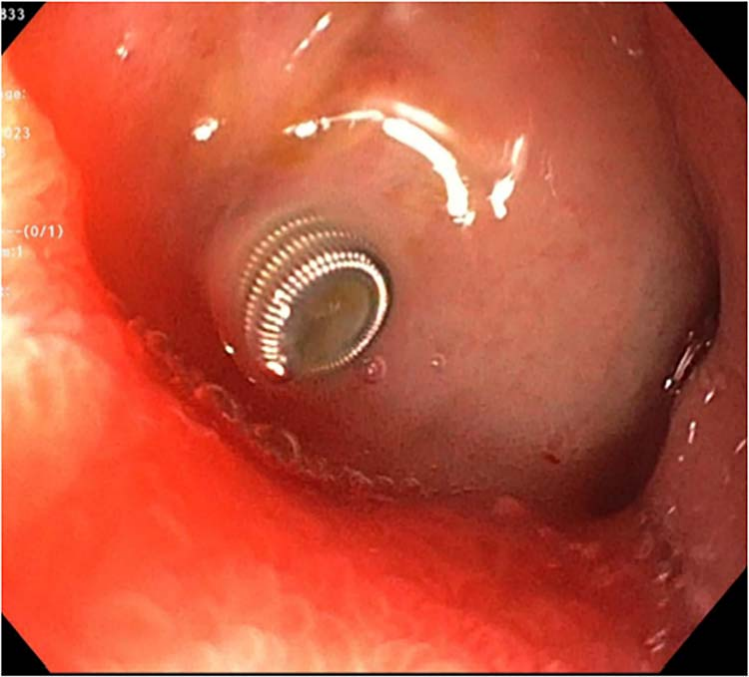
Endoscopic view showing a visible embolization coil at the center of the duodenal ulcer with no active bleeding.

A 72-year-old woman with a history of multiple sclerosis, mixed connective tissue disease on long-term steroid therapy, and osteoarthritis with frequent NSAID use presented to the emergency department with a three-day history of hematemesis, hematochezia, and lightheadedness. She was hypotensive and anemic (hemoglobin 6.1 g/dl, baseline 11 g/dl). Additional laboratory work-up showed a normal platelet count of 257 × 10^3^/ul, normal coagulation profile (prothrombin time 12.5 s, INR 1.18, partial thromboplastin time 29.3 s), and an elevated BUN/Cr of 55.7 (BUN 39 mg/dl, Creatinine 0.7 mg/dl). Esophagogastroduodenoscopy (EGD) performed after volume resuscitation revealed a 10 mm oozing cratered ulcer on the anterior wall of the distal duodenal bulb. Hemostasis was achieved with epinephrine injections and bipolar cautery (Fig. 1), and random gastric biopsies were negative for *H. pylori*. Due to the ulcer’s large size, location at the duodenal sweep, and significant fibrosis, other therapies such as clipping were not possible. However, she required escalation to the intensive care unit 72 h after EGD for new-onset hematochezia with clots and hypotension. CTA abdomen showed massive arterial bleeding in the first and second portions of the duodenum via a branch of the gastroduodenal artery (GDA) (Fig. 2). She underwent angiography which identified a ruptured pseudoaneurysm originating from the retroduodenal artery arising from the GDA with collateral flow through the right gastroepiploic artery which was selectively embolized (Fig. 3). Due to persistently increasing vasopressor requirements, poor transfusion responsive anemia, and acute onset worsening epigastric pain, the patient underwent a second-look EGD three days later, which revealed a protruding 1 mm edge of the previously placed embolization coil at the center of the 10 mm cratered duodenal ulcer with no active bleeding (Fig. 4). Multidisciplinary discussions between gastroenterology, interventional radiology, and surgery determined to pursue maximal acid-suppression therapy and allow the coil to remain in place. Steroid therapy was tapered off and the patient was educated to avoid NSAID use entirely. The patient remained on twice-daily proton pump inhibitor (PPI) therapy with stable hemoglobin and had no additional bleeding events recorded eight weeks after EGD.

## Discussion

Transcatheter arterial embolization (TAE) has revolutionized the management of recurrent peptic ulcer bleeding refractory to standard endoscopic therapies, offering a minimally invasive approach with high efficacy [4]. TAE offers the advantages of lower mortality and complication rates compared to surgery and is the recommended treatment of choice by the American College of Gastroenterology following unsuccessful endoscopic therapeutic interventions [2, 3, 5]. Metallic coils are one of the most commonly used agents for TAE. Other agents include absorbable gelatin sponge particles, N-butyl cyanoacrylate, and polyvinyl alcohol [6]. Arterial coil migration is uncommon with a reported incidence of up to 3% [7]. Visibility of the embolized coil in peptic ulcers after TAE for recurrent bleeding is rare [6]. Complications of coil migration include embolization of adjacent nontarget vessels whilst very rarely distant migration can result in pulmonary embolism, stroke, and myocardial infarction [8]. A rare case of small bowel obstruction from coil migration 6 years after embolization of a gastroduodenal pseudoaneurysm in a patient with hemosuccus pancreaticus is reported in the literature [9]. A single-center study involving regular endoscopic follow-up of 11 patients with visible extravascular coil on endoscopy after TAE for GI bleeding found scar formation in 10 (91%) patients and healing ulceration without bleeding in one (9%) patient on the final look. There was no rebleeding or major complication attributed to TAE or the visible coil during the follow-up duration (mean 123 days; range 5–2093 days) [6]. Factors that contribute to coil migration include premature deployment and utilization of undersized coils, peptic ulcer-induced mucosal fragility, vasospasm from coil irritation, and gastrointestinal peristalsis [6, 7]. There are no established guidelines for the management of this observation. However, conservative management with close monitoring and maximal acid suppression with PPI therapy has been associated with good outcomes [8]. Management should be individualized by a multidisciplinary collaboration between gastroenterology, interventional radiology (IR), and surgery in symptomatic patients.

In the case of this patient, the protruded coil was identified on repeat endoscopy for increasing pressor requirements three days after TAE for recurrent peptic ulcer bleeding. In the absence of active bleeding, conservative management after multidisciplinary consultation with IR and surgery resulted in favorable outcomes eight weeks post-procedure. This case adds to the growing body of literature raising awareness of this phenomenon and supports the conservative management of patients with asymptomatic coil visualized on endoscopy in peptic ulcer after TAE. The concurrent use of corticosteroids with NSAIDs and the absence of gastroprotection were major risk factors for the development of peptic ulcer disease and bleeding in this patient. In a nested case–control study involving 8478 patients, elderly patients 65 years and above who were concurrently taking NSAIDs and corticosteroids were found to have a 15 times greater risk of developing peptic ulcer disease compared to individuals not using either medication [10]. Combination therapy with NSAIDs and steroids, especially in the elderly, should therefore be restricted to urgent, evidence-based indications with plans for prompt steroid tapering as possible. Ulcer prophylaxis with antisecretory therapy is highly recommended [10].
